# Electroacupuncture Ameliorates Neuroinflammation-Mediated Cognitive Deficits through Inhibition of NLRP3 in Presenilin1/2 Conditional Double Knockout Mice

**DOI:** 10.1155/2021/8814616

**Published:** 2021-01-06

**Authors:** Kun Li, Guoqi Shi, Yang Zhao, Yiwen Chen, Jie Gao, Lin Yao, Jiaying Zhao, Hongzhu Li, Ying Xu, Yongjun Chen

**Affiliations:** ^1^Department of Physiology, School of Basic Medicine, Shanghai University of Traditional Chinese Medicine, 1200 Cailun Road, Shanghai 201203, China; ^2^South China Research Center for Acupuncture and Moxibustion, Medical College of Acu-Moxi and Rehabilitation, Guangzhou University of Chinese Medicine, Guangzhou 510006, China; ^3^School of Rehabilitation Science, University of Traditional Chinese Medicine, 1200 Cailun Road, Shanghai 201203, China; ^4^School of Pharmaceutical Sciences, Guangzhou University of Chinese Medicine, Guangzhou 510006, China; ^5^Center for Brain Science and Brain-Inspired Intelligence, Guangdong-Hong Kong-Macao Greater Bay Area, Guangzhou 510515, China; ^6^Guangdong Province Key Laboratory of Psychiatric Disorders, Southern Medical University, Guangzhou 510515, China

## Abstract

Neuroinflammation is considered as one of the crucial pathogenesis in promoting neurodegenerative progress of Alzheimer's disease (AD). As complementary and alternative therapy, electroacupuncture (EA) stimulation has been widely used in clinical practice for anti-inflammation. However, whether EA promotes the cognitive deficits resulting from neuroinflammation in AD remains unclear. In this study, the presenilin 1 and 2 conditional double knockout (PS cDKO) mice, exhibited a series of AD-like pathology, robust neuroinflammatory responses, and memory deficits, were used to evaluate the potential neuroprotective effect of EA at *Baihui* (GV 20) and *Shenting* (GV 24) by behavioral testing, electrophysiology recording, and molecular biology analyzing. First, we observed that EA improved memory deficits and impaired synaptic plasticity. Moreover, EA possesses an ability to suppress the hyperphosphorylated tau and robust elevated NLRP3, ASC, Caspase-1, IL-1*β*, and IL-18 in PS cDKO mice. Importantly, MCC950, a potent and selective inhibitor of NLPR3 inflammasome, has similar effects on inhibiting the hyperphosphorylated tau and the robust elevated NLRP3 components and neuroinflammatory responses of PS cDKO mice as well as EA treatment. Furthermore, EA treatment is not able to further improve the AD-like phenotypes of PS cDKO mice in combination with the MCC950 administration. Therefore, EA stimulation at GV 20 and GV 24 acupoints may be a potential alternative therapy for deterring cognitive deficits in AD through suppression of NLRP3 inflammasome activation.

## 1. Introduction

Alzheimer's disease (AD) is the most common type of dementia in the elderly population [1], characterized by progressive decline in the cognitive and psychomotor function. The extracellular deposition of amyloid-*β* plaques, hyperphosphorylation of tau-associated neurofibrillary tangles, and neuroinflammation caused by the innate immune system in the brain are considered as the major pathological hallmarks of AD and have gained much attention [2–4]. Previous studies show that plagues, tangles, and neuronal debris persistently activate primed microglia, which results in a constant production of proinflammatory mediators, such as interleukin-1 beta (IL-1*β*), tumor necrosis factor alpha (TNF-*α*), nitric oxide, chemokines, and complements [5, 6] These mediators maintain microglial activation and induce neuroinflammatory responses in early phase of AD, which aggravate tau pathology, synaptic, and neuronal dysfunction. With the continuity of neuroinflammation, the neurodegenerative progress of AD also accelerated [7]. Therefore, blockage of neuroinflammation-mediated synaptic and neural dysfunction may be potential therapeutic strategies for preventing the occurrence of AD, delaying its process, or improving its symptoms.

The inflammasome is a critical protein complex of the innate immune system, which consists of pyrin domain-containing protein 3 (NLRP3) receptor, the adaptor apoptosis-associated speck-like protein (ASC), and the cysteine protease caspase-1 [8]. Activation of NLPR3 inflammasome mediates the maturation of caspase-1 and the secretion of proinflammatory cytokines IL-1*β* and IL-18 [9]. Previous research suggested that the role of NLRP3 inflammasome activation by amyloid-beta (A*β*) may be critical for IL-1*β* processing and subsequent inflammatory responses [10, 11]. Recent studies provide evidence that the NLRP3 inflammasome are essential for both the progression of A*β* and tau pathology directly in AD [12, 13]. These findings indicate that NLRP3 inflammasome may be a potential therapeutic target for AD (or neurodegenerative disorders).

As the subunits of *γ*-secretase, the presenilin (PS)1/2 are responsible for the proteolytic processing of amyloid precursor proteins (APPs) and other proteins involved in apoptosis and neuronal adhesion [14]. Previous reports indicated that the mutations in the PS1 and PS2 were discovered in brain of early-onset AD patients [15]. To investigate the therapeutic effect and underlying mechanism in AD, we used the PS1 and PS2 conditional double knockout (PS cDKO) mice as an animal model with deletion of PS1 in the forebrain and PS2 in the whole body [16, 17]. PS cDKO mice displayed obvious AD-like phenotypes, including tau hyperphosphorylation, synaptic and neuronal loss, brain atrophy, and memory deficits, especially glia activation and neuroinflammatory responses but no distinct change in deposition of A*β* plaques [18–20]. Since PS cDKO mice exhibit robust inflammatory responses from young adults (3 months), they have been chosen as a suitable animal model to mimic the progress of AD and study the pharmacological mechanisms of neuroinflammation-mediated neurodegenerative disorders.

As one of the most recognized alternative therapies, acupuncture has been widely used to treat cognitive impairment and neurodegenerative disorders including AD [21, 22]. Both *Baihui* (GV 20) and *Shenting* (GV 24) are located on the midline of the head, which are most commonly used acupoints for the treatment of neurological and psychiatric disorders such as mania, epilepsy, depression, and dementia [23–25]. However, whether EA stimulus can attenuate the cognitive impairment resulting from neuroinflammation in AD remains unclear. Here, we applied EA at GV 20 and GV 24 acupoints to four months old PS cDKO mice for three weeks. Specifically, we studied the effects of EA on behavioral changes, synaptic plasticity, inflammation response, and NLRP3 inflammasome component analyses. We found that EA treatment ameliorated cognitive deficits, impaired long-term potentiation (LTP) induction, and abnormal expression of NMDA receptors of PS cDKO mice, which might be associated with the antineuroinflammatory effects of EA through blockage of the NLRP3 inflammasome signaling pathway in hippocampus. This study suggested that EA at GV20 and GV24 acupoints should be considered as an effective therapeutic strategy against neuroinflammation-mediated neurodegenerative disease.

## 2. Materials and Methods

### 2.1. Animals

The PS cDKO mice were generated and genotyped as previously described [18]. Briefly, the cDKO mice were acquired by crossing the forebrain-specific PS1 heterozygous knockout mice with conventional PS2 heterozygous knockout mice based on the C57BL/6 J genetic background. Mice with the transgene Cre, fPS1/fPS1, and PS2^−/−^ served as PS cDKO mice, and their littermates (Cre−, PS1^+/+^, and PS2^+/+^) served as control wild-type (WT) mice. Mice were group-housed (four per cage) at a temperature of 22 ± 2°C, 40–70% humidity on a 12-h light/dark cycle (the lights were on from 7 a.m. to 7 p.m.), with ad libitum access to food and water. PS cDKO mice and their littermate WT mice at 4 months were used in this study. All the experiments were approved by the Institutional Animal Care and Use Committee at Guangzhou University of Chinese Medicine (GZUCM).

### 2.2. Electroacupuncture and MCC950 Treatment

**Figure 1:**
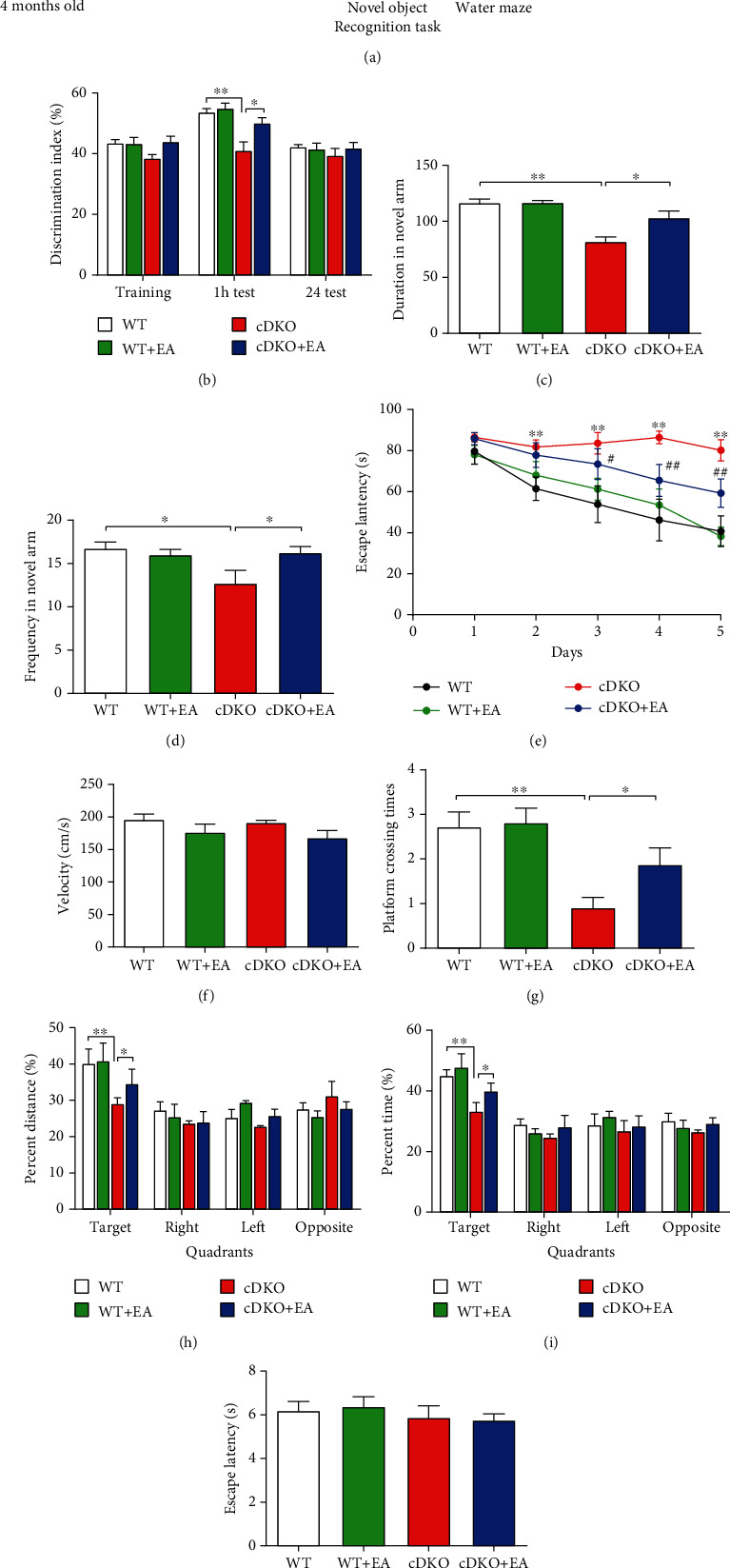
EA treatment ameliorates impaired memory in PS cDKO mice. (a) The experimental timeline of habituation, EA stimulation, and behavioral tests. (b) Effects of EA treatment on recognition memory in PS cDKO mice by NOR task. (c, d) The duration spent and frequency visited in the novel arm of the Y-maze. (e) The escape latency to the hidden platform in target quadrant during the first phase of 5 consecutive training days of the MWM test. (f) Mice of four groups exhibited no differences in swimming speed in the MWM test. (g) Platform crossing times in the second phase, probe trial on day 6 of the MWM test. (h, i) Percentage of distance swim and time spent in the target quadrant during the probe trial of the MWM test. (j) Escape latency to the visible platform without differences among the four groups in the MWM test. (*n* = 8 mice for each group). Data are the mean ± S.E.M., ^∗^*p* < 0.05, ^∗∗^*p* < 0.01 vs WT mice. ^#^*p* < 0.05,^##^*p* < 0.01 vs cDKO mice.

Four-month-old PS cDKO mice and their littermates were randomly divided into four groups: WT, WT+EA, PS cDKO (cDKO), and cDKO+EA. Disposable acupuncture needles (0.16 × 7 mm) were purchased from Beijing Zhongyan Taihe Medical Instrument Co., Ltd (Zhongyan Taihe, Beijing, China). To avoid the side effects of anesthetic, EA stimulation was applied to the mice in EA treatment groups in the awakened state. The self-made fixing device is made of 50 mL epoxy tube and thin insulated iron wire. Enough holes were left on the epoxy tube to make sure mice inside could not move but breathe normally. There was a bigger hole on the top of the mouse head to facilitate the EA stimulation. Mice were fixed in the self-made fixing devices. The needles were inserted to a depth of 4 mm at the *Baihui* (GV 20) and *Shenting* (GV 24) acupoints after the skin had been cleaned with alcohol wipes. Then, the mice were connected to a Master-8 Stimulator (Master-8, AMPI, Israel), and electrical current (2 Hz, 1 mA) was given to the needles. As shown in (Figure 1(a)), mice received EA stimulation for 15 min at 10 : 00 a.m. each day for 5 consecutive days per week, and the whole course of treatment lasted 3 consecutive weeks. To avoid this and minimize the effect, all the mice were put into self-made fixing devices for adaptation two weeks before EA stimulation as described previously [26]. During the three weeks for EA stimulation, mice in the WT group and in the cDKO group were fixed in the same devices in the awakened state and treated the same way without giving EA stimulation.

MCC950 is a kind of NLRP3 inflammasome inhibitor used for the interventional study, and it was purchased from Sigma-Aldrich (St. Louis, MO, USA). Four-month-old mice and their littermates were randomly divided into five groups: wild-type (WT), PS cDKO (cDKO), cDKO + EA, cDKO + MCC950, and cDKO + EA + MCC950. The mice in WT and PS cDKO groups were fed normally, and the mice in cDKO + EA and cDKO + EA + MCC950 groups were given EA treatment for three consecutive weeks as describe above. MCC950 was dissolved in sterile saline. After three weeks of EA treatment, three times of intraperitoneal injection (IP) of MCC950 (20 mg/kg) with or without EA or vehicle were given once every two days. Mice were killed in the next day after the last intraperitoneal injection of MCC950. Hippocampus was dissected on ice and stored at -80°C until being processed to analyze the protein and gene expression.

### 2.3. Behavior Analysis

#### 2.3.1. Novel Object Recognition (NOR) Task

The experiment was performed as we previously described [27], and each mouse was placed in a new object recognition box (40 cm × 40 cm × 25 cm) for 3 days to adapt to the environment. During the training phase, two toys with the same color and shape were placed in the experimental box, and each group of mice was allowed to explore freely for 15 min. In the tests 1 and 24 h after the training, the mice were put back in the same experimental box, and one of the familiar toys was replaced with a new one with different colors and shapes. Mice were allowed to explore two objects freely for 15 min. Noldus software and tracking systems (Noldus, Wageningen, Netherland) were used to record and analyze behavior. The calculation of the preference index is used to evaluate recognition memory: the ratio of the time spent exploring any identical or novel object to the total time spent exploring two objects.

#### 2.3.2. Y-Maze

The Y-maze consists of three identical arms (30 cm × 6 cm × 15 cm), which are arranged in 120° order. In the training phase, a movable baffle is used to seal one arm of the Y-maze as a closed arm, and the other two arms are kept unblocked as a starting arm and an open arm, respectively. First, the mice were placed face to the center of the maze in the starting arm, so that the mice could explore freely in the maze for 8 min, and then the mice were taken out. After 1 h, remove the movable baffle, open the closed arm, place the mouse in the starting arm in the same way again (keep the three arms clear), let the mouse explore freely in the three arms for 8 min, take out the mouse to clean the fecal urine after the observation and record, wipe the maze with 75% alcohol, clear the residual smell, and then continue the record of the next mouse. The spatial memory ability of mice was measured by observing and recording the time of staying in each arm and the times of entering the closed arm.

#### 2.3.3. Morris Water Maze (MWM)

The MWM experiment was used to measure the hippocampal-dependent spatial memory in mice, and the protocol was as described before [28]. The MWM experimental swimming pool has a diameter of 120 cm, a height of 60 cm, and a temperature of 22 ± 1°C. We placed the round platform 10 cm in diameter in the center of the target quadrant and set its height 2 cm below the water surface to hide the platform position. In the training phase, the time of finding the hidden platform in 90 s was recorded for 5 consecutive days, and the position of the hidden platform in the target quadrant remained unchanged during the recording period. On day 6, the space exploration test was carried out, the platform was removed, and the mice were allowed to swim freely in the pool for 90 s. Then, the percentage of time spent in each quadrant and the swimming distance, the frequency of crossing the original position of the platform, and the swimming speed were recorded. The escape latency within 90 s was recorded at the visual platform stage on day 7 to evaluate whether the visual acuity of the mice affected the experiment.

### 2.4. Electrophysiological Recording

#### 2.4.1. Slice Preparation

The experimental scheme was as described in the previous study [29]. In short, we anesthetized the mice with ether then decapitated them. Cut the hippocampus into 400 *μ*m slices and prepared with Vibroslice (Leica VT1000s, Wetzlar, Germany) in ice-cold artificial cerebrospinal fluid (ACSF). ACSF (in mM): 120 NaCl, 2.5 KCl, 2 CaCl_2,_ 2 MgSO_4,_ 1.2 NaH_2_PO_4_, 26 NaHCO_3,_ and 10 D-glucose. After cutting, hippocampal slices were left inside a holding chamber with ASCF at temperature (33 ± 1°C) for recovery for 30 min. Then, hippocampal slices maintained for 2-8 h at room temperature (25 ± 1°C). All the solutions were saturated with 95% O_2_/5% CO_2_ (vol/vol).

#### 2.4.2. Electrophysiology Recordings

The slices were transferred from the holding chamber to a recording chamber with superfusion of ACSF (3 mL/min) at constant 32–33°C. The Schaffer collateral (SC) was stimulated with a two-concentric bipolar stimulating electrode (FHC, Bowdoinham, ME, USA) to evoke the field excitatory postsynaptic potentials (fEPSPs). Monophasic pulse (0.1 ms duration) of the constant current was used to test the stimuli at a frequency of 0.033 Hz, the intensity of which was adjusted to produce 30% of the maximum response. Axon MultiClamp 700B (Molecular Devices, Sunnyvale, CA, USA) amplifier with ACSF filled glass pipettes (1–3 M*Ω*) was put in the stratum radiatum of CA1 to record stable fEPSPs at SC-CA1 synapse for 20 min. Next, high frequency stimulus (HFS, 100 Hz, 1 s) was given to induce LTP, and evoked fEPSPs were recorded for 60 min with the same intensity of the test stimulus. The strength of synaptic transmission could be determined by measuring the initial (10–60% rising phase) slope of the fEPSPs. Clampex version 10.3 software (Axon, US) was applied to get the data. LTP levels were based on the slopes of fEPSPs at an average of 50–60 min after tetanus stimulation, which was normalized to the slopes of the last 10 min fEPSPs before tetanus stimulation.

### 2.5. mRNA Analysis

Quantitative real-time PCR (qRT-PCR) was performed as we described in the previously published paper [30]. In short, after behavioral tests, mice were sacrificed. The hippocampal tissue of mice was dissected and removed and immediately froze in liquid nitrogen. Total RNA of mice from the hippocampus was isolated by using a E.Z.N.A® Total RNA Kit (Omega Bio-Tek, Inc., Norcross, GA). To obtain cDNA, the reversed transcription of total RNA was achieved by using a PrimeScriptTMRT reagent Kit (Takara, Japan). Quantitative PCR with SYBR Green Dye Gene Expression Assays was applied to determine the gene expression by using the ABI7500 system (Applied Biosystems, Carlsbad, CA, USA). The required primers were synthesized by Shanghai Sangon Biological Engineering Technology Company (Shanghai, China). Required primers sequences were as follows: TNF-*α* (forward: 5′-GAAC TGGC AGAA GAGG CACT; reverse: 5′-AGGG TCTG GGCC ATAG AACT), IL-1*β* (forward: 5′-CAGG CAGG CAGT ATCA CTCA; reverse: 5′-AGCT CATA TGGG TCCG ACAG), ASC (forward: 5′-TCCA ACCC CTAA AACT GCGT; reverse: 5′-CACG AACT GCCT GGTA CTGT), IL-18 (forward: 5′-CTGG CTGT GACC CTCT CTGT; reverse: 5′-CTGG AACA CGTT TCTG AAAG), caspase-1 (forward: 5′-TCTC ACCG CTTC GGAC AT; reverse: 5′-ACAT CTGG GACT TCTT CG), *NLRP3* (forward: 5′-AGTG GATG GGTT TGCT GGGA; reverse: 5′-GCGT GTAG CGAC TGTT GAGG), *β*-actin (forward: 5′-AGCCCATGTACGTAGCCATCC; reverse: 5′-TCTCAGCTGTGGTGGTGAAG). The cycle threshold was determined for each sample as the initial increase in fluorescence above background. *β*-Actin was used as internal control for normalization.

### 2.6. Western Blot Analysis

After behavioral tests, mice were sacrificed. The hippocampal tissue of mice was dissected and removed and immediately froze in liquid nitrogen. Homogenized the frozen tissues with tissue lysate in ice-cold RIPA buffer (composition: 50 mM Tris-HCl pH 7.4,150 mM NaCl, 1% Triton X-100, 1 mM EDTA, 1% sodium deoxycholate, 0.1% SDS, 1 mM sodium fluoride, 2 mM sodium orthovanadate) supplemented with 1 mM phenylmethane sulfonyl fluoride and inhibitors of protease and phosphatase (aprotinin, leupeptin, and pepstatin A, 10 *μ*g/mL for each). Centrifugation at 15,000 rpm and 4**°**C for 30 min to obtained the lysates. Electrophoresed 40 *μ*g proteins of tissue lysate on 10% SDS-PAGE gels, then the proteins were transferred to nitrocellulose membrane (Amersham Biosciences, Buckinghamshire, UK). The proteins on the membrane were incubated with primary antibodies at 4**°**C overnight. Washed the membranes three times and then incubated them with HRP-conjugated secondary antibodies (1 : 3000, Cell Signaling Technology, Danvers, MA, USA) at room temperature for 1 h. Then, a SuperSignal West Femto Kit (ThermoFisher Scientific, Waltham, MA, USA) was used to develop the membranes. Image Quant software (Tanon, Shanghai, China) and Image J were applied to scan the films and quantified the intensities of protein bands. The relative protein levels were normalized to *β*-actin. The following primary antibodies were used in the Western blot analyses: rabbit anti-NR1 (1 : 1000, CST), rabbit anti-NR2A (1 : 1000, CST), rabbit anti-NR2B (1 : 1000, Abcam), mouse anti-p-tau (396) (1 : 1000, Santa Cruz, Biotechnology, Dallas, Texas, USA), mouse anti-p-tau (404) (1 : 1000, Santa Cruz), mouse anti-tau (1 : 1000, Santa Cruz), rabbit anti-NLRP3 (1 : 1000, CST), mouse anti-ASC (1 : 1000, Santa Cruz), mouse anti-caspase-1 (1 : 1000, Santa Cruz), and anti-*β*-actin (1 : 1000, CST).

### 2.7. Statistical Analysis

All the data were presented as the mean ± standard error of mean (S.E.M) and were analyzed by using SPSS 21.0 (Chicago, IL, USA) and GraphPad Prism version 5.0 (San Diego, CA, USA). One-way or two-way ANOVA with post hoc Bonferroni's multiple comparison tests were used to analyze the results.*p* < 0.05 was considered statistically significant. When carrying out data analyses, the experimenters were blind to the grouping of mice.

## 3. Results

### 3.1. EA Treatment Ameliorates Memory Deficits in PS cDKO Mice

According to previous studies, 5 months old PS cDKO mice have exhibited severe impaired recognition and spatial memory [18]. Thus, NOR, Y-maze, and MWM test were performed to assess the effects of EA on impaired memory in PS cDKO mice. The experimental timeline of behavioral tests is shown in Figure 1(a). NOR is usually used to analyze the hippocampus-dependent recognition memory function. During the training session of NOR, the mice from four groups showed no difference in their preference for two same objects. After an hour, we found that PS cDKO mice spent less time exploring new objects than the WT mice in the retention test. However, EA treatment enhanced the preference degree and recognitive index of new objects in the PS cDKO mice (Figure 1(b)). Thus, our experimental results indicated that EA treatment could improve the short-term recognition memory deficits in PS cDKO mice.

The Y-maze is an effective way to measure the spatial memory ability of animals by using the nature of mice to explore new and different environments. As shown in Figures 1(c) and 1(d), we observed that PS cDKO mice displayed lower preference for new arms compared with WT mice. However, EA treatment significantly increased the duration (Figure 1(c)) and frequency (Figure 1(d)) of entering the new arm in PS cDKO mice, suggesting that EA could improve the spatial memory deficits in PS cDKO mice.

Subsequently, the MWM test were conducted to evaluate the spatial reference memory of animals. During the first phase (5 consecutive days) for training, the escape latency of PS cDKO mice was longer compared with WT mice, indicating that PS cDKO mice might spend more time finding the hidden platform. However, EA treatment significantly shortened their escape latency time in PS cDKO mice (Figure 1(e)). In the second phase (day 6), the probe trial was carried out to measure the memory retention of these mice in the target quadrant. There were also no significant differences in their swimming velocities among four groups (Figure 1(f)). It was found that the WT group showed a strong preference in the target quadrant, but the PS cDKO group exhibited no discrimination in the distance and time occupancies among the four quadrants, and they crossed fewer times in the target quadrant comparing to other quadrants. Afterwards, PS cDKO mice with EA treatment spent longer time, crossed more times, and swam further in target quadrant. In the meantime, when comparing the duration of stay in other quadrants, there were no differences among the four groups (Figures 1(g)–1(i)). During the second phase, day 7, mice in four groups exhibited no differences in the visible platform test (Figure 1(j)), indicating that EA treatment can improve the spatial reference memory deficits of PS cDKO mice, but has no impact on their sensorimotor abilities. The above results suggest that EA treatment may have positive therapeutic effect on ameliorating impaired recognition and spatial memory in the PS cDKO mice.

### 3.2. EA Treatment Improves Impaired Synaptic Plasticity in the Hippocampus of PS cDKO Mice

**Figure 2:**
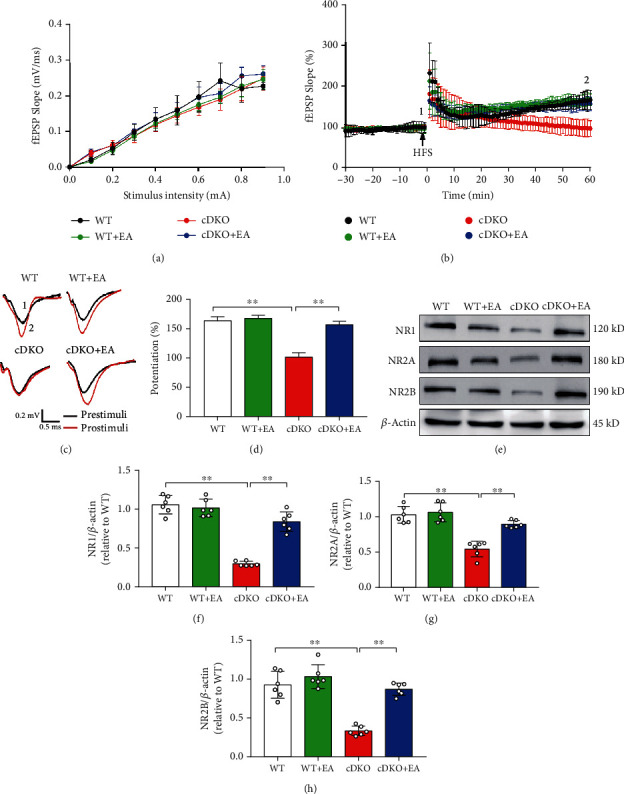
EA treatment improves impaired LTP at SC-CA1 synapses and level of NMDA receptors in the hippocampus of PS cDKO mice. (a) Quantitative data of I/O curves obtained at different stimulus intensities. (b) Normalized fEPSP slopes in SC-CA1 synapses from hippocampal slices of indicated mice. (c) Representative fEPSP traces taken before (1, black lines) and 60 min after tetanus stimulation (2, red lines) from each group. Scale bar: 0.5 mV, 10 ms. (d) Quantitative data of potentiation at 60 min after tetanus stimulation (*n* = 7 slices from 5 mice for each group). (e) Representative Western blot of synapse-associated proteins in the hippocampus. (f)–(h) Quantification of Western blot of the synapse-associated proteins in the hippocampus (*n* = 6 mice for each group). The levels of synapse-associated proteins were standardized based on the respective level of *β*-actin. The values were expressed as relative changes to the respective WT mice, which was set to 1. Data are the mean ± S.E.M., ^∗^*p* < 0.05, ^∗∗^*p* < 0.01.

Since synaptic plasticity may be one of the basic underlying mechanisms for improving ability of learning and memory, we observed the effects of EA treatment on alternation of LTP induction by determining fEPSPs at SC-CA1 synapses of PS cDKO mice. As shown in Figure 2(a), their slopes under different stimulation intensities were similar among four groups, suggesting that EA treatment had no impact on basal synaptic transmission at SC-CA1 synapse of PS cDKO mice. Likewise, the magnitude of LTP induced by HFS in PS cDKO mice was also decreased expressively in comparison with WT mice, in accordance with our previous study [27]. Interestingly, the decreased LTP magnitude induced by HFS was ameliorated by EA treatment (Figures 2(b)–2(d)). Taken together, these results manifest that EA treatment can moderate the impaired synaptic plasticity in PS cDKO mice.

The role of NMDA receptors in the LTP induction at SC-CA1 synapses in the hippocampus is well established. We next determine whether EA treatment can recover their deficiency, which are known as playing key roles in synaptic plasticity and memory of PS cDKO mice in our previous study [27]. Western blot analysis showed that expression levels of NR1, NR2A, and NR2B in the hippocampus of PS cDKO mice were decreased when compared to WT mice, while EA treatment reversed those (Figures 2(e)–2(h)). The above results suggest that EA treatment reverse the downregulation in the expression of synaptic plasticity-associated proteins involved in the neuropathology of PS cDKO mice.

### 3.3. Hyperphosphorylated Tau and Neuroinflammatory Responses of PS cDKO Mice Are Reduced by EA Treatment

**Figure 3:**
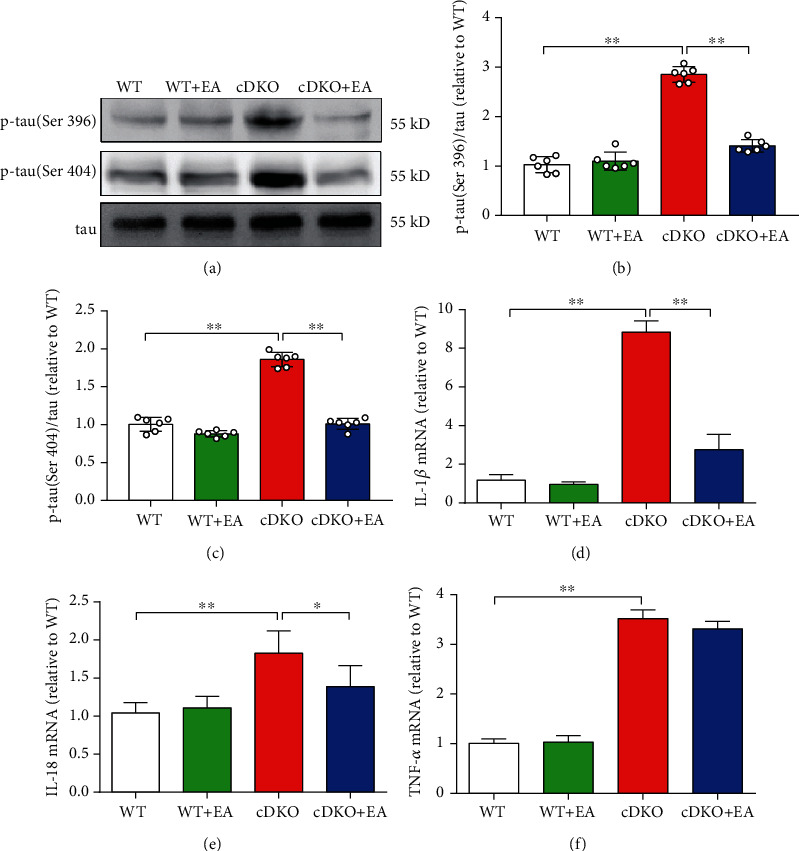
EA treatment inhibits tau hyperphosphorylation and elevated inflammatory response in the hippocampus of PS cDKO mice. (a) Representative Western blot of phosphor-tau (Ser396/Ser404) in the hippocampus. (b, c) Quantification of Western blot for p-tau in the hippocampus (*n* = 6 mice for each group). The protein levels of p-tau were normalized with the levels of their respective total tau. The values were expressed as relative changes to the respective WT mice, which was set to 1. (d)–(f) Quantitative mRNA levels of IL-1*β*, IL-18, and TNF-*α* in the hippocampus by qRT-PCR (*n* = 5 mice for each group). The mRNA levels were standardized based on the respective level of *β*-actin. Values were expressed as relative changes to WT mice, which was set to 1. Data are the mean ± S.E.M., ^∗^*p* < 0.05, ^∗∗^*p* < 0.01.

Previous research has shown that tau hyperphosphorylation and neuroinflammatory responses are linked with synaptic plasticity and memory dysfunction in AD [31, 32]. Firstly, we assessed the effect of EA treatment on the expression of hyperphosphorylated tau in the hippocampus of PS cDKO mice. The Western blot analysis showed that there was obvious elevation in the expression levels of phosphorylated tau (Ser396/Ser404) in the hippocampus of PS cDKO mice, which could be reversed by EA treatment (Figures 3(a)–3(c)). Moreover, we also determined the effects of EA treatment on the mRNA levels of proinflammatory cytokines in the hippocampus of PS cDKO mice, such as IL-1*β*, IL-18, and TNF-*α* by qRT-PCR analyses. The results showed that the mRNA levels of IL-1*β*, IL-18, and TNF-*α* were significantly upregulated in the hippocampus of PS cDKO mice. Notably, EA treatment could suppress the mRNA levels of IL-1 *β* and IL-18 (Figures 3(d) and 3(e)), but no effect on the TNF-*α* mRNA expression (Figure 3(f)). These results above indicate that EA treatment has the ability to exert neuroprotective effects on synaptic plasticity and memory deficits in PS cDKO mice by inhibiting hyperphosphorylated tau and some proinflammatory cytokines in hippocampus.

### 3.4. EA Treatment Reverses the Robust Upregulated Levels of NLRP3 Inflammasome in the Hippocampus of PS cDKO Mice

**Figure 4:**
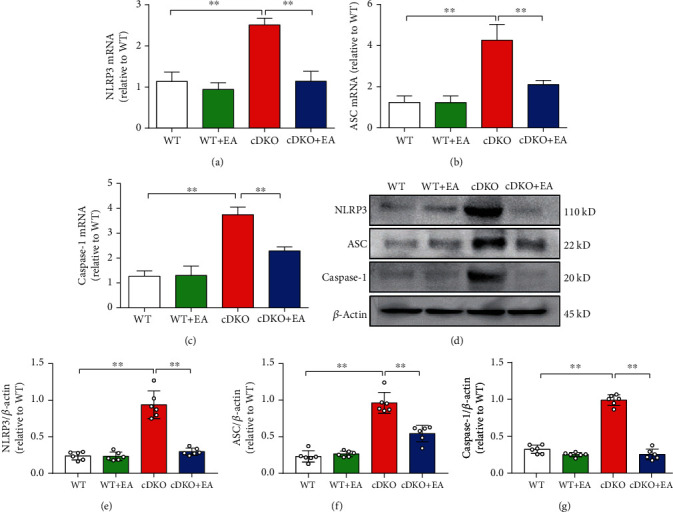
EA treatment reverses the robust upregulated levels of NLRP3 inflammasome in the hippocampus of PS cDKO mice. (a)–(c) Total RNA was isolated and subjected to quantitate the mRNA levels of NLRP3, ASC, and caspase-1 in the hip by qRT-PCR (*n* = 5 mice for each group). The mRNA levels were standardized based on the respective level of *β*-actin. Values were expressed as relative changes to WT mice, which was set to 1. (d) Representative Western blot of NLRP3, ASC, and caspase-1 in the hippocampus. (e)–(g) Quantification of Western blot for NLRP3, ASC, and caspase-1 in the hippocampus (*n* = 6 mice for each group). The protein levels of NLRP3, ASC, and caspase-1 were normalized with the levels of *β*-actin. The values were expressed as relative changes to the respective WT mice, which was set to 1. Data are the mean ± S.E.M., ^∗^*p* < 0.05, ^∗∗^*p* < 0.01.

Inflammatory corpuscles are innate immune system sensors, which can regulate the activation of microglia to induce inflammation according to risk signals. In this process, NLRP3 inflammasome is activated by double stimulations, which leads to the heterogenesis of ASC and the activation of caspase-1 [8, 9]. Here, we analyzed the mRNA expression of NLRP3, ASC, and caspase-1. The results showed that EA treatment significantly inhibited the mRNA level of these inflammasome-related proteins in the hippocampus of PS cDKO mice (Figures 4(a)–4(c)). Similarly, Western blot analysis showed that the expression of NLRP3, ASC, and caspase-1 was significantly increased in the hippocampus of PS cDKO mice, while EA treatment significantly inhibited the expression of NLRP3 and ASC (Figures 4(d)–4(g)).

### 3.5. EA Treatment Inhibits the Neuroinflammatory Response in the Hippocampus of PS cDKO Mice through Inhibiting NLRP3 Inflammasome

**Figure 5:**
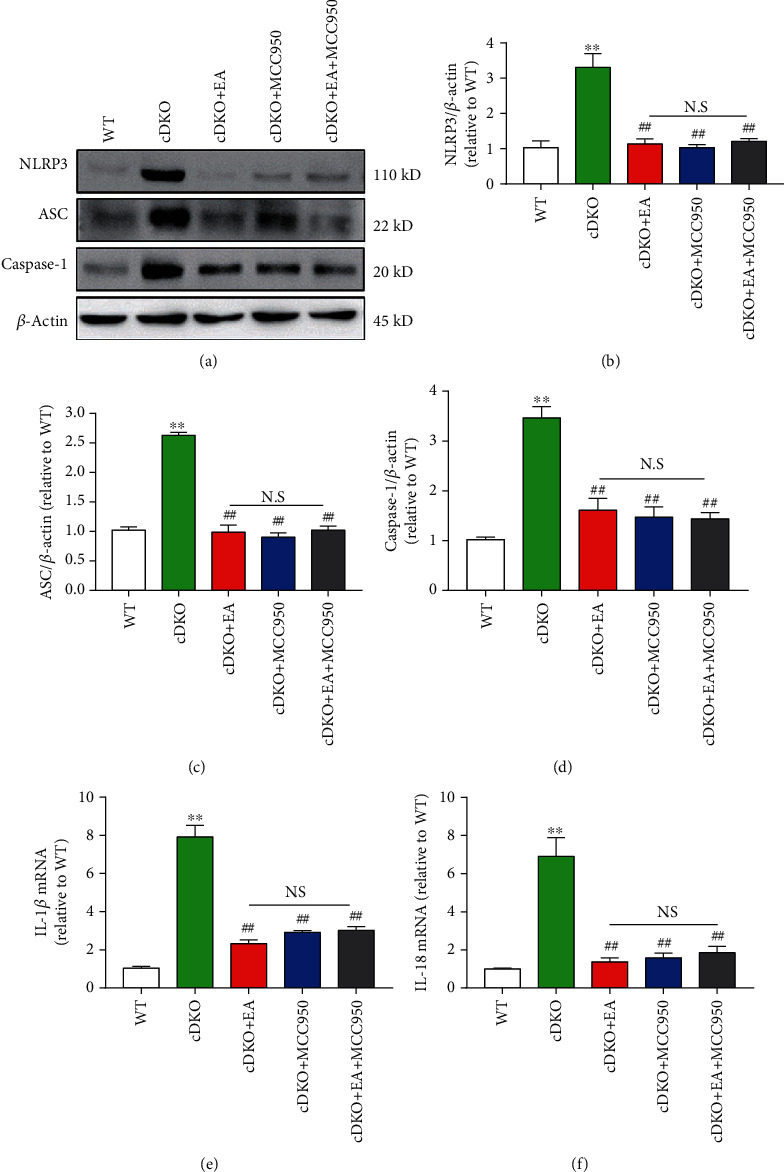
MCC950 does not further increase the effect of EA on inhibiting NLRP3 inflammasome and neuroinflammatory responses in the hippocampus of PS cDKO mice. (a) Representative Western blot of NLRP3, ASC, and caspase-1 in the hippocampus. (b)–(d) Quantitative analysis of NLRP3, ASC, and caspase-1 in the hippocampus were standardized based on the respective level of *β*-actin (*n* = 6 mice for each group). (e, f) Total RNA was isolated and subjected to quantitate the mRNA levels of IL-1*β* and IL-18 in the hippocampus by qRT-PCR (*n* = 5 mice for each group). The mRNA levels were standardized based on the respective level of *β*-actin. Values were expressed as relative changes to WT mice, which was set to 1.0. Data are the mean ± S.E.M., ^∗^*p* < 0.05, ^∗∗^*p* < 0.01 vs WT mice. ^#^*p* < 0.05, ^##^*p* < 0.01 vs cDKO mice.

In order to better understand the molecular mechanism of cognitive and memory deficits in PS cDKO mice, we thus studied the effect of EA treatment on neuroinflammation, using MCC950, a NLRP3 inhibitor, to inhibit the expression of NLRP3 in the hippocampus of PS cDKO mice. Western blot analysis showed that the protein expression of NLRP3, ASC, and caspase-1 in the hippocampus could be significantly reduced by MCC950 treatment alone (Figures 5(a)–5(d)). Meanwhile, MCC950 also significantly reduced the mRNA levels of IL-1*β* and IL-18. Strikingly, we found that EA and MCC950 treatment exhibited similar effects on the level of IL-1*β*, IL-18, NLRP3, ASC, and caspase-1 (Figures 5(a)–5(f)). In order to determine whether they work in the same way, we combined EA and MCC950 treatment for PS cDKO mice. The experimental results showed that the combined treatment did not further reduce the expression of NLRP3 (Figures 5(a)–5(d)) and the mRNA levels of IL-1*β* and IL-18 (Figure 5(e) and 5(f)) in the hippocampus of PS cDKO mice. Together, these findings provide further evidence that EA treatment may inhibit neuroinflammatory responses by blocking the NLRP3-ASC signaling pathway.

### 3.6. EA Treatment Ameliorates Tau Pathology and NMDA Receptor Damage of PS cDKO Mice through Inhibiting NLPR3 Inflammasome

**Figure 6:**
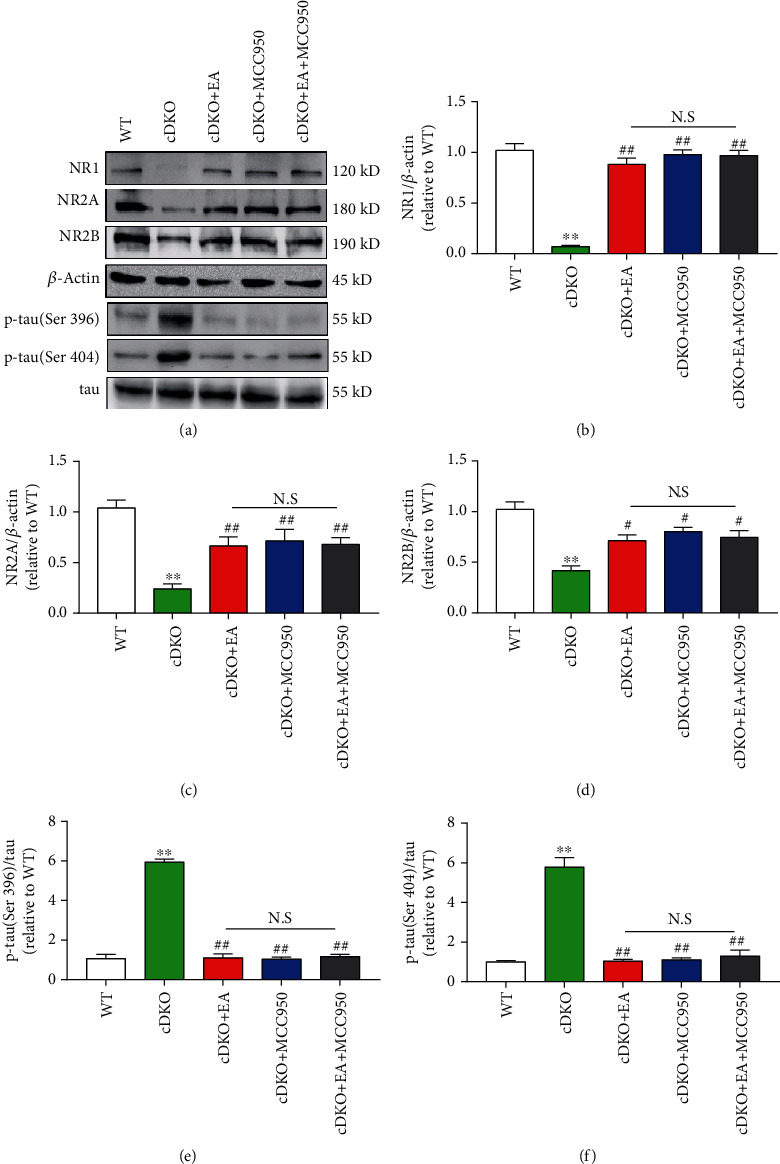
MCC950 does not further increase the effect of EA on ameliorated hyperphosphorylated tau and impaired NMDA receptors in the hippocampus of PS cDKO mice. (a) Representative Western blot of NR1, NR2A, NR2B, p-tau (Ser396), and p-tau (Se404) in the hippocampus. (b)–(f) Quantification of NR1, NR2A, NR2B, p-tau (Ser396), and p-tau (Ser404) in the hippocampus according to the respective levels of *β*-actin or total tau (*n* = 6 mice for each group). The values were expressed as relative changes to the respective WT mice, which was set to 1. Data are the mean ± S.E.M., ^∗^*p* < 0.05, ^∗∗^*p* < 0.01 vs WT mice. ^#^*p* < 0.05, ^##^*p* < 0.01 vs cDKO mice.

To further investigate the correlation between EA treatment, NLRP3 inflammation, and AD-like phenotypes, Western blot was performed to evaluate the effects of EA, MCC950, and combination of two treatments. As shown in Figure 6, both EA treatment and MCC950 treatment alone could significantly rescue synaptic proteins in the hippocampus PS cDKO mice, including NR1, NR2A, and NR2B. In addition, the increase of phosphorylated tau levels of Ser396 and Ser404 was significantly inhibited by MCC950 or EA treatment alone. However, we found that the combined therapy did not further improve the NMDA receptor function damage in the hippocampus of PS cDKO mice (Figures 6(b)–6(d)) or further reduce the phosphorylated tau level of Ser396 and Ser404 (Figures 6(e) and 6(f)). These results further indicate that EA has the neuroprotective effect on PS cDKO mice through inhibiting NLRP3 inflammasome, reducing hyperphosphorylated tau and improving the expression of NMDA receptors.

## 4. Discussion

In this study, we found that 3 weeks EA stimulation at GV 20 and GV 24 acupoints could effectively ameliorate the memory deficits, improve the expression of NMDA receptors and LTP induction, inhibit hyperphosphorylated tau, and robust elevated neuroinflammatory responses in the PS cDKO mice. Furthermore, EA treatment reversed the robust upregulated levels of NLRP3 inflammasome in the hippocampus of PS cDKO mice, and the MCC950 administration did not further increase the effects of EA treatment on inhibiting the upregulated levels of NLRP3 inflammasome in the hippocampus of PS cDKO mice.

Our results showed that PS cDKO mice in 5 months old exhibited significant memory deficits in NOR, Y-maze, and MWM test, which was consistent with previous studies [28, 33]. Furthermore, EA stimulation at GV 20 and GV 24 acupoints could effectively ameliorate the cognitive deficits of PS cDKO mice (Figure 1). Consistent with our result, previous study proved that EA treatment could improve learning-memory ability of the AD mice model and ameliorate poststroke cognitive impairments via inhibition periinfarct astroglia and microglial/macrophage P2 purinoceptors-mediated neuroinflammation [34–36]. In addition, PS cDKO mice exhibited obvious deficits in LTP induction at SC-CA1 synapses, and impaired magnitude of LTP in PS cDKO mice was ameliorated by EA treatment (Figure 2). Similarly, our previous study proved the efficacy of EA at GV 20 and GV 24 on promoting LTP induction and cognitive deficits in rat exposed to maternal separation [37]. Other studies also proved that EA stimulation could improve the spatial learning and memory of AD animal models induced by ethanol or vessel occlusion [38–40]. Clinically, EA or acupuncture therapy has been widely used to treat cognitive impairment of neurodegenerative disorders including AD and dementia [41, 42]. Both results of AD animal models and clinical practice support the efficacy of EA on treating cognitive deficits.

The ionotropic receptors of glutamate NMDA receptors are spread in postsynaptic membranes of the hippocampus, and NMDA receptor stimulation elicits the translocation of CaMKII to postsynaptic sites, where CaMKII is activated by NMDAR-triggered calcium influx and plays an important role in LTP induction and memory formation [35, 43]. In our study, Western blot analysis showed that the expression levels of NR1, NR2A, and NR2B in the hippocampus of PS cDKO mice were all increased after EA treatment (Figure 3). Similarly, our previous study has proved that these PS cDKO mice exhibited the impaired expression of NMDA receptors and dramatic decrease in the NMDAR/AMPAR ratio in comparison with WT mice [27]. Consistent with our results, one recent study indicated that EA at GV 20 and GV 24 acupoints improved increased intracellular calcium concentrations regulated by the activation of NMDA receptors [44]. Taken together, these results provide further evidence that restoration of the NMDA receptors in PS cDKO mice by EA at GV 20 and GV 24, which may relate to the deficits of the cognitive function and synaptic plasticity in PS cDKO mice.

Besides of significant age-related AD-like symptoms, previous studies showed that PS cDKO mice exhibited a series of pathology, such as elevated hyperphosphorylated tau [18, 27, 33, 45]. Abnormal phosphorylation of tau, the major component of the neurofibrillary tangle, has been associated with synaptic plasticity and memory deficits in AD [32]. We found that there was an obvious hyperphosphorylation of tau at Ser396 and Ser404 in the hippocampus of PS cDKO mice, and the hyperphosphorylated tau in the hippocampus was reversed after the EA treatment (Figure 3). These results above indicate that EA treatment can protect neurons in the hippocampus of PS cDKO mice by lowering the hyperphosphorylation of tau. Consistent with our results, recent evidence suggested that EA could counteract diabetes-associated tau hyperphosphorylation in adult rat and had a possible beneficial effect on the brain cholinergic system in diabetes [46]. These results agree with our observations in this study, which suggests that EA stimulation at GV 20 and GV24 ameliorates learning and cognitive deficit in PS cDKO mice via lowering the tau pathology.

Neuroinflammatory is also critical in progression of AD [47]. Except for hyperphosphorylated tau and impaired synaptic plasticity, PS cDKO mice showed the increased mRNA levels of IL-1*β* and TNF-*α* (Figure 3), which are consistent with our previous studies [27]. Strikingly, this study is the first to show that PS cDKO caused the robust to increase in the expression of NLRP3, ASC, and caspase-1 at both the protein and mRNA levels (Figure 3). Consistent with our results, the levels of NLRP3 inflammasome are substantially elevated both in brains from AD patients [10] and several rodent AD models [10, 12, 13, 48], since previous studies have shown that NLRP3 inflammasome activation induces the cleavages of cytokine precursors to generate active IL-1*β* and IL-18 [9]. In addition, NLRP3 inflammasome was also been found to drive the tau pathology in a recent study [13]. Therefore, it is likely that our study provides a strong correlation between hyperphosphorylated tau, neuroinflammation, and NLRP3 inflammation in PS cDKO mice, which is in accordance with accumulated evidences that NLRP3 inflammasome has been implicated in the pathogenesis of AD [49].

Meaningfully, our studies exhibited that EA treatment significantly inhibited the robust elevated levels of components and products of NLRP3 inflammasome components in PS cDKO mice (Figures 4 and 5). Specially, we provide further evidence that inhibiting NLRP3 inflammasome by MCC950 reduces the robust elevated tau, IL-1*β*, and IL-18 as well as NLRP3 components (Figures 5 and 6). Intriguingly, we found that EA at GV20 and GV24 acupoints has similar effect on NLRP3 inflammation components and neuroinflammation responses as MCC950 administration (Figures 5 and 6). In accordance with our results, other studies found that using both EA and manual acupuncture could ameliorate the upregulation of NLRP3 inflammasome as well as their synaptic and cognitive dysfunction in senescence-accelerated (SAMP8) mice [50, 51]. Furthermore, our results demonstrated that EA treatment fail to further enhance the inhibiting effect of MCC950 on NLRP3 components and products (Figures 5 and 6). Together, these results provide further support to the idea that EA suppresses the IL-1*β* and IL-18 via inhibiting the NLRP3 inflammasome. It is conceivable that the findings of this study could shed light on the mechanisms of EA therapy on the neuroinflammatory-medicated cognitive impairment in AD.

## Data Availability

The data used to support the findings of this study are available from the corresponding author upon request.
